# Neurodevelopment of children who are HIV‐exposed and uninfected in Kenya

**DOI:** 10.1002/jia2.26149

**Published:** 2023-11-01

**Authors:** Michelle A. Bulterys, Irene Njuguna, Maureen King'e, Daisy Chebet, Hellen Moraa, Laurén Gomez, Alvin Onyango, Kenneth Malavi, Gladys Nzia, Martin Chege, Jillian Neary, Anjuli D. Wagner, Kendall A. Lawley, Dalton Wamalwa, Sarah Benki‐Nugent, Grace John‐Stewart

**Affiliations:** ^1^ Department of Global Health University of Washington Seattle Washington USA; ^2^ Department of Epidemiology University of Washington Seattle Washington USA; ^3^ Kenyatta National Hospital Nairobi Kenya; ^4^ Department of Pediatrics and Child Health University of Nairobi Nairobi Kenya; ^5^ Departments of Pediatrics, Medicine University of Washington Seattle Washington USA; ^6^ Department of Medicine University of Washington Seattle Washington USA

**Keywords:** neurodevelopment, HIV exposure, children who are HIV‐exposed uninfected, CHEU, maternal mental health, intimate partner violence

## Abstract

**Introduction:**

Predictors of neurodevelopment among children who are HIV‐exposed uninfected (CHEU) are poorly understood.

**Methods:**

Mothers with and without HIV and their children were enrolled during 6‐week postnatal care visits across seven sites in Kenya between March 2021 and June 2022. Infant neurodevelopment was assessed using the Malawi Developmental Assessment Tool, including social, language, fine motor and gross motor domains. We used multivariate linear mixed effects models to identify associations between 1‐year neurodevelopment scores, HIV and antiretroviral therapy (ART) exposures, and household factors, adjusted for potential confounders and clustered by the site.

**Results:**

At 1‐year evaluation, CHEU (*n* = 709) and children who are HIV‐unexposed uninfected (CHUU) (*n* = 715) had comparable median age (52 weeks) and sex distribution (49% vs. 52% female). Mothers living with HIV were older (31 vs. 27 years), had lower education (50% vs. 26% primary) and were more likely to be report moderate‐to‐severe food insecurity (26% vs. 9%) (*p* < 0.01 for all). Compared to CHUU, CHEU had higher language scores (adjusted coeff: 0.23, 95% CI: 0.06, 0.39) and comparable social, fine and gross motor scores. Among all children, preterm birth was associated with lower gross motor scores (adjusted coeff: −1.38, 95% CI: −2.05, −0.71), food insecurity was associated with lower social scores (adjusted coeff: −0.37, 95% CI: −0.73, −0.01) and maternal report of intimate partner violence (IPV) was associated with lower fine motor (adjusted coeff: −0.76, 95% CI: −1.40, −0.13) and gross motor scores (adjusted coeff: −1.07, 95% CI: −1.81, −0.33). Among CHEU, *in utero* efavirenz (EFV) exposure during pregnancy was associated with lower gross motor scores compared to dolutegravir (DTG) exposure (adjusted coeff: −0.51, 95% CI: −1.01, −0.03). Lower fine and gross motor scores were also associated with having a single or widowed mother (adjusted coeff: −0.45, 95% CI: −0.87, −0.03) or a deceased or absent father (adjusted coeff: −0.81, 95% CI: −1.58, −0.05), respectively.

**Conclusions:**

Biologic and social factors were associated with child neurodevelopment. Despite socio‐demographic differences between CHEU and CHUU, 1‐year neurodevelopment was similar. Addressing IPV and food insecurity may provide benefits regardless of maternal HIV status. DTG use was associated with higher neurodevelopmental scores in CHEU, compared to EFV regimens, potentially contributing to a lack of neurodevelopmental difference between CHEU and CHUU.

## 1 INTRODUCTION

Successful prevention of vertical transmission programmes over the last decade has contributed to a rapidly growing population of almost 16 million children who are HIV‐exposed uninfected (CHEU) around the world, with an additional one million born every year in sub‐Saharan Africa (SSA) [1, 2]. Compared to children who are HIV‐unexposed uninfected (CHUU), CHEU are at increased risk of morbidity, adverse birth outcomes, growth faltering, environmental and pathogenic exposures, poor mental health and social inequities as a member of a family affected by HIV [2, 3, 4, 5, 6, 7, 8, 9, 10]. In multiple high HIV burden countries in SSA, the population of CHEU accounts for over 20% of all children under 15 years of age, and the SSA region is estimated to have the largest proportion of children under 5 years of age at risk of not meeting their developmental potential [11, 12]. Early neurodevelopmental delays are associated with poorer physical and mental health and learning potential [11]. A child's neurodevelopment is remarkably sensitive to parental caregiving and home environment factors, especially during the first 1000 days of life. It is possible to reverse early delays in children, and the earlier the intervention, the greater the impact [13, 14].

Most, but not all, studies have found an increased risk of neurodevelopmental delays in CHEU compared to their CHUU peers. Previous studies have been limited by small sample sizes. Preterm birth, *in utero* ART exposure, maternal viremia and early child inflammatory markers have been associated with significantly poorer neurodevelopment among CHEU [15, 16, 17]. Studies have noted differences in language, social and motor skills, brain composition and structure, and altered cell‐mediated immunity between CHEU and CHUU [9, 18, 19]. While biologic aetiologies of neurodevelopmental outcomes among CHEU have been assessed, there are fewer data on modifiable, social and behavioural factors that may synergistically influence neurodevelopmental outcomes. Women living with HIV are especially vulnerable to poverty, parental relationship instability, low paternal involvement, intimate partner violence (IPV) and poor maternal mental health; such factors may influence their ability to care responsively for their children and promote healthy neurodevelopment [12, 20, 21, 22, 23, 24, 25, 26, 27, 28, 29].

Kenya alone is home to nearly one million CHEU for whom research is urgently needed to identify caregiver and home factors to reduce the risk of suboptimal child neurodevelopment [1]. In 2016, the World Health Organisation (WHO) recommended dolutegravir (DTG), an integrase strand transfer inhibitor, as first‐line treatment for all adolescents and adults living with HIV, and in 2019, for women of reproductive age [30]. Prior to DTG scale‐up, non‐nucleoside reverse transcriptase inhibitor regimens containing efavirenz (EFV) were commonly used during pregnancy. *In utero* exposure to EFV‐based regimens has been associated with neurodevelopmental deficits among 2‐year‐old CHEU in Botswana, compared to non‐EFV‐based regimens [31]. DTG‐based regimens and EFV‐based regimens have comparable safety in pregnancy [32]; however, there are few data on the impact of *in utero* DTG exposure on neurodevelopmental outcomes of CHEU. This study aimed to assess the associations between caregiver and household factors, HIV and ART exposure, and child neurodevelopment in Kenya.

## 2 METHODS

### 2.1 Study overview

The HOPE Study is an ongoing prospective longitudinal cohort in Kenya aimed to understand the impacts of HIV and ART exposure on infant health and development. Mothers living with and without HIV were recruited with their infants (1000 CHEU and 1000 CHUU) at 4–10 weeks of age during routine postnatal care at seven maternal and child health clinics across the Nairobi and Western Kenya regions between March 2021 and June 2022. Mother‐infant pairs are being followed every 6 months until children reach 3 years. This exploratory analysis identified cofactors associated with child neurodevelopment among the subset of children who have reached age 1 year and had complete neurodevelopmental assessments by June 2023 (Figure S1).

### 2.2 Data collection

#### 2.2.1 Outcome ascertainment

To measure child neurodevelopment, we administered the Malawi Developmental Assessment Tool (MDAT) [33], a validated test designed specifically for the SSA cultural context. The MDAT assessment generates scores for four domains, social, language, fine motor and gross motor, with 36–42 pass/fail items in each. Scripts for each item were translated and back‐translated to Kiswahili and Dholou. Tests were administered by a trained assessor in the preferred language of the mother/caregiver and combined direct child observation and caregiver reporting. Once a child reached six consecutively failed items in a domain, the assessors moved on to the subsequent domains until all were complete. Assessments that were either incomplete (e.g. due to noisy environment, fussy child, assessor still completing training) or invalid (e.g. the assessment did not note six consecutive fails) were excluded from this analysis (Figure S1). Assessors underwent a rigorous training curriculum and each conducted ≥10 supervised practice assessments prior to certification and study start. We utilised a train‐the‐trainer approach involving six half‐day didactic and practical sessions over the course of several weeks. Certified trainers then employed a similar training to all study nurses and routinely reviewed in‐person and over video recordings to ensure consistency. MDAT scores were assessed as raw continuous scores per domain.

#### 2.2.2 Exposures ascertainment

A primary exposure of interest was maternal HIV status (CHEU vs. CHUU). Per national Kenyan guidelines, all pregnant women presenting with unknown HIV status are tested for HIV. Maternal HIV status was confirmed by self‐report and review of medical records. Women living with and without HIV were enrolled into this study when their infants reached approximately 6 weeks of age. At health facilities, infants of children born to women living with HIV are tested for HIV DNA on dried blood spots at 6 weeks, 6 and 12 months and then tested using an HIV antibody test at 18 months. For this study, infant HIV status was abstracted from medical records at each time point to ensure CHEU remained HIV negative. A small subset of the 709 CHEU (*n* = 34) included in this analysis still had pending HIV test results by the time of this analysis. Other exposure variables collected at baseline (6 weeks postpartum) included maternal socio‐demographic information, family characteristics, medical history and household factors. The gestational age of children was ascertained by maternal report and antenatal care medical records, and preterm birth was defined as less than 37 weeks of gestation.

The following mental health assessments were conducted: the 9‐item Patient Health Questionnaire (PHQ‐9, ≥10 cut‐off) [34] for clinically significant, moderate‐to‐severe depression, 10‐item Kessler Psychological Distress Scale (K10, ≥20 cut‐off) [35, 36] for anxiety, the Multidimensional Scale of Perceived Social Support (MSPSS, ≤35 = low, 36–60 = medium, ≥61 = high) and the Hurt‐Insult‐Threaten‐Scream (HITS, ≥10 cut‐off) [37] for IPV. The degree of household food insecurity was assessed using the Household Hunger Scale [38]. Existing referral pathways in each of the participating clinics were identified for study nurses to refer caregivers and/or infants for psychiatric, IPV, child neurodevelopmental or nutritional support. Neurodevelopmental referrals were informed by failed “red flag” items on MDAT assessments. Additionally, mothers living with HIV were asked questions regarding HIV, experience with status disclosure to their partner, and ART initiation, duration and regimen. Data on maternal ART regimens were abstracted from medical records, including information on any regimen switches during or after pregnancy. The final models assessed the most recently prescribed regimen during pregnancy and compared mothers who most recently received DTG‐based regimens compared to EFV‐based regimens. We additionally compared the subset women who received DTG‐only to those who received EFV‐only.

### 2.3 Data analysis

Descriptive statistics and univariable log‐binomial models described differences between CHEU and CHUU (Table 1). Univariable and multivariable linear mixed effects models determined associations between neurodevelopment scores, HIV‐exposure status and caregiver factors, adjusting for confounders selected *a priori*. We expected some degree of site‐specific differences, as sites spanned across Nairobi and Western Kenya, and therefore, we included facility as a random intercept in all models. Mean MDAT scores at 1 year of age were compared between CHEU and CHUU, and scores were compared among all children to test for associations with caregiver cofactors. Potential confounders adjusted in multivariable analyses included preterm birth, maternal age (years), education level, marital status, and infant sex and age (weeks). Based on the literature, we expected these factors to be associated with exposures, such as maternal HIV status, and child neurodevelopment scores. Collinearity was examined using a threshold of 10% change in standard error and multivariable models included non‐collinear variables univariately associated with neurodevelopment (*p* < 0.05). Exposures evaluated in the models included maternal depression, anxiety, distress (depression and/or anxiety), marital status, IPV, household food insecurity and absence of a biologic father (defined as either deceased or uninvolved in any way in the child's life, including physically, financially and emotionally). Among CHEU, MDAT scores were compared by maternal ART start timing (pre/post‐pregnancy), HIV disclosure to partner (ever/never) and ART regimen (DTG, EFV or protease inhibitor [PI]‐based).

**Table 1:** Socio‐demographic characteristics comparing CHEU and CHUU

Characteristic: *n* (%) or median (interquartile range)	Overall *N* = 1424	CHEU *n* = 709	CHUU *n* = 715	Unadjusted *p*‐value
Region				
Nairobi	588 (41%)	272 (38%)	316 (44%)	ref
W Kenya	835 (59%)	437 (62%)	398 (56%)	**0.03**
Child sex is female	723 (51%)	348 (49%)	375 (52%)	0.2
Child age (weeks)	52 (52, 52)	52 (52, 53)	52 (52, 52)	0.6
Preterm birth (gestational age <37 weeks)	39 (2.7%)	21 (3.0%)	18 (2.5%)	0.6
Child is orphaned (maternally, paternally or both)	109 (7.7%)	71 (10%)	38 (5.3%)	**<0.001**
Exclusively breastfed at 6 weeks	1381 (97%)	695 (98%)	686 (96%)	**0.005**
Number of times breastfed in last 24 hours	14.0 (11.0, 16.0)	14.0 (12.0, 18.0)	14.0 (10.0, 16.0)	**<0.001**
Child has siblings	1126 (79%)	622 (88%)	504 (71%)	**<0.001**
Mother age (years)	29.0 (25.0, 33.0)	31.0 (27.0, 35.0)	27.0 (24.0, 31.0)	**<0.001**
Mother education, primary or less	540 (38%)	352 (50%)	188 (26%)	**<0.001**
Mother is employed (professionally or casually)	279 (20%)	142 (20%)	137 (19%)	0.7
Mother marital status				
Married (monogamous)	1112 (78%)	517 (73%)	595 (83%)	ref
Married (polygamous)	109 (7.7%)	79 (11%)	30 (4.2%)	**<0.001**
Steady partner	57 (4.0%)	22 (3.1%)	35 (4.9%)	0.2
Single, separated or widowed	144 (10%)	91 (13%)	53 (7.4%)	**<0.001**
Mother height (cm)	162 (158, 167)	162 (157, 167)	162 (158, 167)	0.2
Mother body mass index <18.5	41 (2.9%)	27 (3.8%)	14 (2.0%)	**0.04**
Moderate‐to‐severe household food insecurity	246 (17%)	181 (26%)	65 (9.1%)	**<0.001**
Moderate‐to‐severe maternal depression	47 (3.4%)	21 (3.1%)	26 (3.6%)	0.4
Moderate‐to‐severe maternal anxiety	113 (8.1%)	68 (9.9%)	45 (6.3%)	0.5
Moderate‐to‐severe maternal distress	135 (9.6%)	76 (11%)	59 (8.3%)	**0.01**
Maternal report of intimate partner violence	33 (2.4%)	14 (2.0%)	19 (2.7%)	0.1
Perceived level of social support				
High	972 (69%)	446 (63%)	526 (74%)	Ref
Medium	401 (28%)	227 (32%)	174 (24%)	**<0.001**
Low	45 (3.2%)	32 (4.5%)	13 (1.8%)	**<0.001**

*Note*: Table 1 presents basic child and maternal socio‐demographic characteristics among the overall cohort of children (*n* = 1424), as well as comparing children who are HIV‐exposed uninfected (CHEU, *n* = 709) and children who are HIV‐unexposed uninfected (CHUU, *n* = 715). The unadjusted *p*‐value indicates whether there was a statistically significant difference in each characteristic between the two comparison groups using a chi‐squared test.

Unadjusted *p*‐values that indicate statistical significance at the *p* < 0.05 level are bolded.

Abbreviations: CHEU, children who are HIV‐exposed uninfected; CHUU, children who are HIV‐unexposed uninfected.

### 2.4 Ethical board approvals

The study was approved by the University of Washington's Institutional Review Board and the Kenyatta National Hospital's Ethical Review Committee. All participants provided written informed consent.

## 3 RESULTS

### 3.1 Study population

#### 3.1.1 All children (*N* = 1424)

This analysis used data collected from 709 CHEU and 715 CHUU mother‐child pairs who had complete MDAT data. Compared to CHUU, CHEU had a comparable median age at 1‐year neurodevelopmental assessment (52 weeks), proportion born preterm (3% each) and sex distribution (49% vs. 52% female) (Table 1). A greater proportion of CHEU had a father who was either deceased or absent from the child's life (10% vs. 5%). At baseline, mothers living with HIV were more likely to be older (31 vs. 27 years), with only primary school education (50% vs. 26%), have other children (88% vs. 71%), either single or widowed (13% vs. 7%) or in a polygamous marriage (11% vs. 4%), and report moderate‐to‐severe food insecurity (26% vs. 9%, *p* < 0.01 for all). Prevalence of depression, anxiety and IPV were similar between mothers with and without HIV.

#### 3.1.2 CHEU only (*n* = 709)

Among mothers living with HIV, all were on ART; 88% started ART pre‐pregnancy, and 12% post‐pregnancy (Table 2). At baseline, 88% of mothers had already disclosed their HIV status to their primary partner and had been taking ART for a median of 54 months (Interquartile Range [IQR]: 22–89). Of 608 mothers with data on ART regimen during pregnancy, the most recently used regimen during pregnancy was DTG‐based (74%), followed by EFV‐based (20%) and PI‐based regimens (6%). Overall, 30% of mothers switched their ART regimen during pregnancy, and 66% of them had switched from EFV to DTG‐based regimens. Table S1 summarises ART changes during pregnancy and the median duration of ART use.

**Table 2:** HIV and ART‐related characteristics of CHEU population

Characteristic: *n* (%) or median (interquartile range)	*n* = 709
Child received ART prophylaxis by 6 weeks	693 (98%)
Child ART regimen	
AZT based	329 (47%)
NVP alone	364 (53%)
*Unknown*	*16*
Maternal ART start timing	
Pre‐pregnancy	590 (88%)
Post‐pregnancy	83 (12%)
*Unknown*	*36*
Most recent maternal ART regimen during pregnancy	
DTG based	448 (74%)
EFV based	122 (20%)
PI based or other	38 (6.3%)
*Unknown*	*101*
Mother switched ART regimen during pregnancy	191 (30%)
*Unknown*	*67*
Mother ART regimen changes during pregnancy	
DTG only	316 (52%)
DTG from EFV	129 (21%)
EFV only	110 (18%)
EFV from DTG	12 (2.0%)
Other regimens	38 (6.3%)
*Unknown*	*104*
Maternal duration on ART (months)	54.1 (22.2, 88.7)
*Unknown*	*131*
Disclosed HIV status to partner	622 (88%)

*Note*: Table 2 presents data on child and maternal HIV and ART‐related characteristics among the subset of children who are HIV‐exposed uninfected (CHEU) in this cohort (*n* = 709).

Abbreviations: ART, antiretroviral therapy; AZT, azithromycin; DTG, dolutegravir; EFV, efavirenz; NVP, nevirapine; PI, protease inhibitor.

### 3.2 Cofactors of child neurodevelopment

#### 3.2.1 MDAT score comparison for CHEU versus CHUU

Overall, the CHEU and CHUU groups had comparable 1‐year neurodevelopment scores across all domains, in both univariable and multivariable mixed linear effects models with site clustering and adjustment for infant age, sex, preterm birth, and maternal age, education and marital status (Table 3). CHEU exhibited statistically higher language scores than CHUU (adjusted coeff: 0.23, 95% CI: 0.06, 0.39, *p* < 0.01).

**Table 3:** Cofactors of MDAT scores at 1 year among overall cohort (CHEU and CHUU)

	Social, adjusted coeff (95% CI)	*p*	Language, adjusted coeff (95% CI)	*p*	Fine motor, adjusted coeff (95% CI)	*p*	Gross motor, adjusted coeff (95% CI)	*p*
***ENTIRE COHORT—CHEU versus CHUU***								
CHEU (ref: CHUU)—Unadjusted	−0.02 (−0.29, 0.25)	0.88	**0.21 (0.06, 0.37)**	**<0.01**	−0.01 (−0.20, 0.19)	0.96	0.15 (−0.08, 0.37)	0.20
CHEU (ref: CHUU)—Adjusted**^a^**	0.07 (−0.22, 0.36)	0.64	**0.23 (0.06, 0.39)**	**<0.01**	0.02 (−0.19, 0.23)	0.86	0.12 (−0.12, 0.36)	0.33
***ENTIRE COHORT^—^adjusting for CHEU status*** **^b^**								
***Child sex is male (ref: female)***	−**0.46 (**−**0.72,** −**0.19)**	**<0.01**	−**0.18 (**−**0.33,** −**0.02)**	**0.02**	0.06 (−0.13, 0.25)	0.52	0.10 (−0.12, 0.32)	0.37
***Child was born preterm***	−0.29 (−1.10, 0.51)	0.47	−0.09 (−0.55, 0.38)	0.71	−0.23 (−0.80, 0.35)	0.44	−**1.38 (**−**2.05,** −**0.71)**	**<0.001**
***Maternal mental health at 6 weeks postpartum***								
Moderate/severe depression (PHQ‐9 score ≥10)	−0.42 (−1.17, 0.32)	0.26	−0.02 (−0.45, 0.41)	0.92	−0.09 (−0.63, 0.44)	0.74	0.08 (−0.54, 0.70)	0.80
Moderate/severe anxiety (K10 score ≥20)	0.27 (−0.22, 0.77)	0.28	0.04 (−0.25, 0.32)	0.79	0.00 (−0.35, 0.35)	0.99	−0.33 (−0.74, 0.08)	0.11
Level of perceived social support (ref: High)	Ref		Ref		Ref		Ref	
Medium	−0.21 (−0.52, 0.09)	0.17	0.04 (−0.13, 0.22)	0.65	−0.09 (−0.30, 0.13)	0.44	−**0.31 (**−**0.56,** −**0.06)**	**0.02**
Low	−0.12 (−0.88, 0.64)	0.76	0.07 (−0.37, 0.50)	0.77	0.43 (−0.12, 0.97)	0.12	−0.09 (−0.72, 0.54)	0.78
***Family factors at 6 weeks postpartum***								
Deceased or absent biologic father	0.15 (−0.35, 0.65)	0.56	−0.16 (−0.45, 0.12)	0.26	−0.27 (−0.63, 0.08)	0.13	−0.02 (−0.44, 0.39)	0.91
Intimate partner violence (HITS score ≥10)	−0.08 (−0.96, 0.80)	0.86	0.28 (−0.23, 0.79)	0.28	−**0.76 (**−**1.40,** −**0.13)**	**0.02**	−**1.07 (**−**1.81,** −**0.33)**	**<0.01**
Moderate to severe household food insecurity	−**0.37 (**−**0.73,** −**0.01)**	**0.047**	−0.06 (−0.27, 0.15)	0.58	0.01 (−0.25, 0.27)	0.96	−0.21 (−0.51, 0.09)	0.17
Marital status (ref: Married—monogamous)	Ref		Ref		Ref		Ref	
Married—polygamous	−0.26 (−0.76, 0.25)	0.32	0.01 (−0.28, 0.30)	0.92	−0.12 (−0.48, 0.24)	0.52	0.25 (−0.17, 0.67)	0.25
Steady partner, not married	−0.40 (−1.08, 0.27)	0.25	−0.07 (−0.46, 0.32)	0.72	−0.32 (−0.80, 0.16)	0.20	−0.06 (−0.62, 0.50)	0.84
Single or widowed	−0.19 (−0.63, 0.25)	0.40	−0.10 (−0.35, 0.16)	0.46	−0.21 (−0.53, 0.11)	0.20	−0.02 (−0.39, 0.35)	0.90

*Note*: Table 3 presents results from multivariate mixed effects linear models testing for associations between multiple factors and child neurodevelopmental scores at 1 year of age across four domains (social, language, fine motor and gross motor), among the overall cohort. The first models compared domain scores between CHEU and CHUU, unadjusted and then adjusted for infant sex, age, preterm birth, and maternal age, education and marital status; subsequent models adjusted for CHEU status, infant sex, age and preterm birth. Bolded results highlighted in green represent statistically significant findings.

Abbrevitaions: CHEU, children who are HIV‐exposed uninfected; CI, confidence interval; CHUU, children who are HIV‐unexposed uninfected; HITS, Hurt, Insult, Threaten, Scream Assessment Tool; K10, Kessler Psychological Distress Scale; MDAT, Malawi Developmental Assessment Tool; PHQ‐9, Patient Health Questionnaire.

^a^ Multivariable mixed effects linear models adjusted for infant age and sex, preterm birth, and maternal age and marital status.

^b^ Multivariable mixed effects linear models adjusted for infant age and sex, preterm birth and maternal HIV status (child is CHEU vs. CHUU).

#### 3.2.2 Entire cohort, adjusting for CHEU status

**Figure 1:**
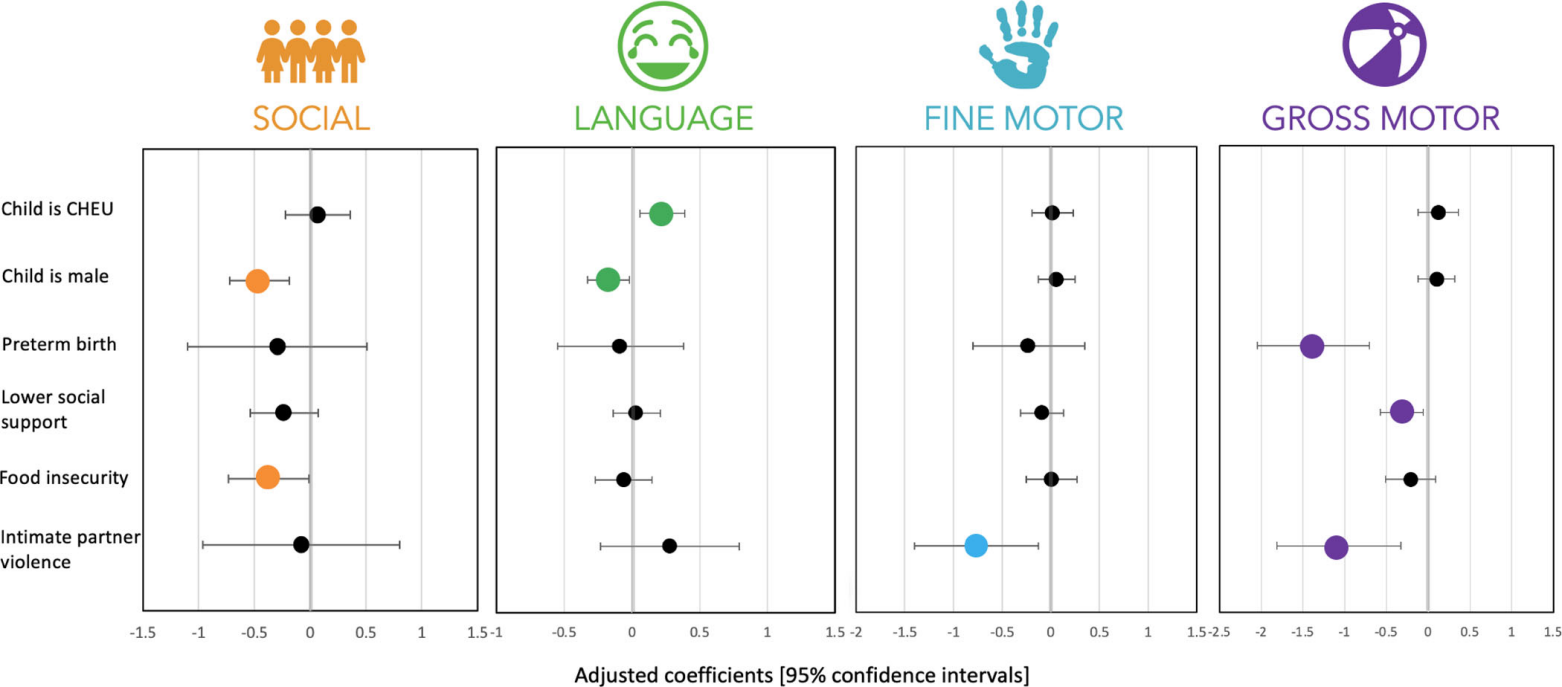
Forest plot of statistically significant cofactors of neurodevelopment among all children. These forest plots present important cofactors of neurodevelopmental scores within the four tested neurodevelopmental domains (social, language, fine motor and gross motor), among all children in this cohort. The small black dots in the forest plots represent the adjusted coefficients with 95% confidence intervals, within each neurodevelopmental domain, and the coloured dots represent the statistically significant findings. Abbreviation: CHEU, children who are HIV‐exposed uninfected.

Compared to female children, male children scored significantly lower in the social (adjusted coeff: −0.46, 95% CI: −0.72, −0.19, *p* < 0.01) and language domains (adjusted coeff: −0.18, 95% CI: −0.33, −0.02, *p* = 0.02) (Table 3 and Figure 1). Children born preterm scored lower in gross motor than children born full‐term (adjusted coeff: −1.38, 95% CI: −2.05, −0.71, *p* < 0.001). IPV was significantly associated with lower fine motor (adjusted coeff: −0.76, 95% CI: −1.40, −0.13, *p* = 0.02) and gross motor scores (adjusted coeff: −1.07, 95% CI: −1.81, −0.33, *p* < 0.01). Moderate‐to‐severe food insecurity was associated with lower social scores (adjusted coeff: −0.37, 95% CI: −0.73, −0.01, *p* = 0.047). Lower gross motor scores were associated with lower levels of maternal perceived social support (adjusted coeff: −0.31, 95% CI: −0.56, −0.06, *p* = 0.02), but not with other mental health measures.

#### 3.2.3 CHEU only

**Figure 2:**
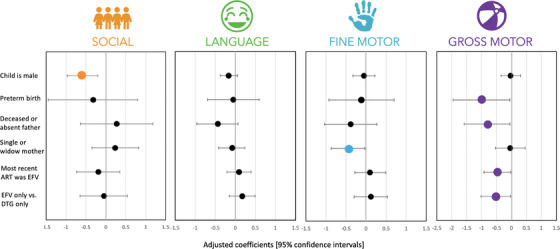
Forest plot of statistically significant cofactors of neurodevelopment, among CHEU only. ART, antiretroviral therapy; CHEU, child who is HIV‐exposed uninfected; DTG, dolutegravir; EFV, efavirenz. These forest plots present important cofactors of neurodevelopmental scores within the four tested neurodevelopmental domains (social, language, fine motor and gross motor), among the subset of CHEU only. The small black dots in the forest plots represent the adjusted coefficients with 95% confidence intervals, within each neurodevelopmental domain, and the coloured dots represent the statistically significant findings. Abbreviation: CHEU, children who are HIV‐exposed uninfected.

Consistent with analyses among all children, male CHEU scored significantly lower than female CHEU in the social domain (adjusted coeff: −0.58, 95% CI: −0.97, −0.20, *p* < 0.01) and CHEU born preterm scored significantly lower in the gross motor domains compared to CHEU born full‐term (adjusted coeff: −1.00, 95% CI: −1.95, −0.05, *p* = 0.04) (Table 4 and Figure 2). Lower gross motor scores were observed among CHEU with deceased or absent fathers (adjusted coeff: −0.81, 95% CI: −1.58, −0.05, *p* = 0.04) and lower fine motor scores were observed among CHEU with single or widowed mothers (adjusted coeff: −0.45, 95% CI: −0.87, −0.03, *p* = 0.04), compared to CHEU with monogamously married mothers.

**Table 4:** Cofactors of MDAT scores among CHEU alone

	Social, adjusted coeff (95% CI)	*p*	Language, adjusted coeff (95% CI)	*p*	Fine motor, adjusted coeff (95% CI)	*p*	Gross motor, adjusted coeff (95% CI)	*p*
*Child sex is male* (ref: female)^a^	−**0.58 (**−**0.97,** −**0.20)**	<**0.01**	−0.17 (−0.38, 0.05)	0.14	−0.05 (−0.33, 0.22)	0.71	−0.03 (−0.35, 0.30)	0.88
***Child was born preterm*** **^a^**	−0.32 (−1.45, 0.80)	0.57	−0.05 (−0.69, 0.59)	0.88	−0.12 (−0.93, 0.70)	0.78	−**1.00 (**−**1.95,** −**0.05)**	**0.04**
***Maternal mental health at 6 weeks postpartum*** **^a^**								
Moderate/severe depression (PHQ‐9 score ≥10)	0.05 (−1.11, 1.20)	0.94	0.02 (−0.63, 0.68)	0.94	−0.08 (−0.92, 0.77)	0.86	−0.50 (−1.48, 0.48)	0.32
Moderate/severe anxiety (K10 score ≥20)	0.33 (−0.32, 0.97)	0.32	−0.13 (−0.49, 0.24)	0.50	−0.07 (−0.53, 0.40)	0.79	−0.19 (−0.74, 0.36)	0.49
Level of perceived social support (ref: High)	Ref		Ref		Ref		Ref	
Medium	−0.24 (−0.66, 0.18)	0.26	0.07 (−0.17, 0.31)	0.57	−0.17 (−0.47, 0.14)	0.29	−0.26 (−0.61, 0.10)	0.16
Low	−0.16 (−1.09, 0.76)	0.73	−0.07 (−0.60, 0.45)	0.69	0.10 (−0.57, 0.78)	0.76	−0.43 (−1.21, 0.36)	0.29
***Family factors at 6 weeks postpartum*** **^a^**								
Deceased or absent father	0.27 (−0.64, 1.18)	0.56	−0.44 (−0.96, 0.07)	0.09	−0.38 (−1.04, 0.27)	0.26	−**0.81 (**−**1.58,** −**0.05)**	**0.04**
Intimate partner violence (HITS score ≥10)	−0.05 (−1.43, 1.34)	0.95	0.44 (−0.35, 1.23)	0.28	−0.08 (−1.09, 0.93)	0.87	−0.96 (−2.13, 0.23)	0.11
Marital status (ref: Married—monogamous)	Ref		Ref		Ref		Ref	
Married—polygamous	0.03 (−0.58, 0.64)	0.92	0.16 (−0.19, 0.51)	0.37	−0.26 (−0.71, 0.18)	0.25	0.00 (−0.52, 0.51)	0.99
Steady partner, not married	−0.67 (−1.80, 0.45)	0.24	−0.22 (−0.86, 0.41)	0.50	−0.46 (−1.28, 0.36)	0.27	−0.72 (−1.67, 0.23)	0.14
Single or widowed	0.23 (−0.35, 0.82)	0.43	−0.08 (−0.41 0.23)	0.63	−**0.45 (**−**0.87,** −**0.03)**	**0.04**	−0.04 (−0.52, 0.46)	0.89
Moderate to severe household food insecurity	−0.37 (−0.82, 0.08)	0.11	0.03 (−0.22, 0.28)	0.83	0.02 (−0.31, 0.35)	0.91	−0.11 (−0.49, 0.27)	0.56
***Maternal ART characteristics*** **^a^**								
Disclosed HIV status by 6 weeks postpartum	0.13 (−0.52, 0.78)	0.69	0.03 (−0.34, 0.40)	0.88	−0.02 (−0.49, 0.45)	0.94	0.23 (−0.32, 0.77)	0.42
Mother started ART post‐pregnancy (ref: pre‐pregnancy)	0.05 (−0.56, 0.67)	0.88	0.21 (−0.13, 0.56)	0.23	0.26 (−0.18, 0.71)	0.25	0.38 (−0.13, 0.91)	0.15
Duration on ART (months)	0.00 (−0.00, 0.01)	0.78	−0.00 (−0.00, 0.00)	0.59	−0.00 (−0.00, 0.00)	0.61	−0.00 (−0.01, 0.00)	0.20
Most recently prescribed ART regimen during pregnancy (ref: DTG based)	Ref		Ref		Ref		Ref	
EFV based	−0.19 (−0.74, 0.35)	0.50	0.09 (−0.21, 0.39)	0.56	0.11 (−0.28, 0.49)	0.59	−**0.47 (**−**0.92,** −**0.02)**	**0.045**
PI based	0.45 (−0.39, 1.29)	0.29	0.11 (−0.34, 0.56)	0.64	0.16 (−0.43, 0.75)	0.60	0.02 (−0.68, 0.72)	0.96
ART changes during pregnancy (ref: DTG only)	Ref		Ref		Ref		Ref	
DTG, switched from EFV	0.04 (−0.49, 0.58)	0.87	0.26 (−0.03, 0.55)	0.08	−0.20 (−0.58, 0.17)	0.29	−0.26 (−0.71, 0.18)	0.25
EFV only	−0.05 (−0.65, 0.54)	0.86	0.18 (−0.14 0.50)	0.29	0.12 (−0.30, 0.54)	0.57	−**0.52 (**−**1.01,** −**0.03)**	**0.04**
EFV, switched from DTG	−0.99 (−2.44, 0.45)	0.18	0.06 (−0.72, 0.85)	0.87	−0.44 (−1.46, 0.59)	0.41	−0.76 (−1.97, 0.45)	0.22
Other regimens	0.48 (−0.37, 1.33)	0.27	0.18 (−0.28, 0.64)	0.44	0.12 (−0.48, 0.72)	0.71	−0.06 (−0.78, 0.65)	0.87

*Note*: Table 4 presents results from multivariate mixed effects linear models testing for associations between multiple factors and child neurodevelopmental scores at 1 year of age across four domains (social, language, fine motor and gross motor), among subset of CHEU only. Models adjusted for infant sex, age, preterm birth, and maternal age, education and marital status. Bolded text in the model results represents statistically significant findings.

Abbreviations: ART, antiretroviral therapy; CHUU, children who are HIV‐unexposed uninfected; CI, confidence interval; DTG, dolutegravir; EFV, efavirenz; HITS, Hurt, Insult, Threaten, Scream Assessment Tool; K10, Kessler Psychological Distress Scale; MDAT, Malawi Developmental Assessment Tool; PHQ‐9, Patient Health Questionnaire; PI, protease inhibitor.

^a^ Multivariable mixed effects linear models adjusted for infant age and sex, preterm birth, and maternal age, education and marital status.

Neurodevelopment was significantly associated with the maternal ART regimen used during pregnancy. Gross motor scores were significantly lower with most‐recent *in utero* exposure to EFV‐based regimens than DTG‐based regimens (adjusted coeff: −0.47, 95% CI: −0.92, −0.02, *p* = 0.04). This finding remained the same in sensitivity analyses comparing CHEU exposed exclusively to EFV to those exposed exclusively to DTG (Table 4), as well as in analyses restricted to full‐term infants only (data not shown).

## 4 DISCUSSION

In this cohort of 1‐year‐old children, we found that CHEU and CHUU had comparable neurodevelopment scores and, unexpectedly, CHEU had higher scores in the language domain. Among all children, lower child neurodevelopment scores were associated with male sex, preterm birth, lower perceived level of social support, and maternal report of IPV and food insecurity. Among CHEU only, lower child neurodevelopment scores were also associated with having a deceased or absent father, single or widowed mother and *in utero* exposure to EFV‐based ART regimens.

Reassuringly, CHEU in this study had similar neurodevelopmental scores to their HIV‐unexposed peers which could be explained by more recent universal test and treat guidelines for people living with HIV and newer, improved ART regimens using DTG‐based combinations. We observed that CHEU had higher language scores, a surprising finding, given that other studies have found a higher risk of language and motor skill delays in CHEU. It is possible that having more siblings (as seen in CHEU) could impact language development, but this finding warrants further investigation. Neurodevelopmental differences between CHEU and CHUU in other studies have been linked to altered brain composition and structure, immune function, adverse birth outcomes and growth faltering [9, 16, 18, 19, 39, 40, 41, 42]. In an extensive meta‐analysis of 21 studies comparing neurodevelopment between CHEU and CHUU under 5 years, 57% of studies found subtle delays among CHEU in at least one domain, and primarily in the language and gross motor domains [40]. Included studies had relatively small sample sizes, and relied on studies published prior to May 2020 prior to newer HIV treatment guidelines.

The effects of ART regimens on CHEU neurodevelopment have been studied with mixed results [40]. To the best of our knowledge, our study is the first to assess CHEU neurodevelopment in a largely DTG‐exposed cohort. DTG has superior efficacy, less frequent drug resistance and comparable safety during pregnancy compared to EFV‐based regimens [32]. EFV‐based regimens have been associated with a higher risk of microcephaly and other neurologic disorders among CHEU [43]. We found that *in utero* EFV‐exposed CHEU scored significantly lower in gross motor than DTG‐exposed CHEU, even when accounting for regimen switches during pregnancy. A study in Botswana among 2‐year‐old CHEU found that *in utero* EFV exposure was associated with significantly poorer language and motor skills, compared to exposure to non‐EFV‐regimens (abacavir/ZDV/lamivudine or PI‐based regimens) [31]. Moreover, EFV‐exposed children had longer ART exposure than children exposed to other regimens, and longer EFV exposure was associated with more pronounced deficits [31]. In our study, women on EFZ had longer ART duration than those on DTG and a higher likelihood of pre‐pregnancy ART use (Table S1). To disentangle the potential collinearity of duration and EFZ exposure, we compared EFZ and DTG in a subset of women with pre‐pregnancy use of ART (Table S2). In this analysis, the association of EFZ with lower gross motor scores persisted, albeit not significantly, likely due to the much smaller sample. In addition, in the overall CHEU cohort, the duration of ART was not associated with lower gross motor scores. Together, these findings suggest that the effect may be related to EFZ exposure rather than ART duration. Despite WHO recommendation, DTG uptake has remained suboptimal in SSA among reproductive‐aged women, partly due to initial concerns around neural tube defects [44]. Over time, the vast majority of mothers living with HIV will be on DTG‐based regimens, making it impossible to discern the impact of DTG‐based regimens. Our study was conducted in the opportune period of changing regimen implementation, providing a unique opportunity to examine the impact of different regimens.

Maternal marital status, absence of a biologic father, IPV and food insecurity were significantly associated with lower child neurodevelopment scores. Parental relationship conflict and household violence can threaten a child's neurodevelopment [22, 45, 46, 47, 48, 49, 50, 51]. Over a quarter of women of reproductive age in eastern Africa were estimated to have experienced IPV in the past year [26, 52, 53]; the prevalence of IPV among Kenyan pregnant women is estimated to be approximately 10%, with almost all perpetrators being a current or former husband or partner [54]. Among Kenyan women, previous experiences with IPV were strongly predictive of incident IPV during pregnancy and postpartum, and could serve as an important screening tool for women at increased risk of IPV [54, 55]. A large meta‐analysis of psychological therapies for women experiencing IPV concluded that individualised counselling and therapy was beneficial. The standard of care for Kenyan women living with HIV includes referrals to IPV counsellors; however, barriers to successful referral include disclosure of IPV and low rates of referral uptake [56]. A randomised controlled trial tested an intervention package, which included IPV‐centred training for HIV care providers, an on‐site IPV counsellor for immediate support, and pictorial take‐home materials for clients, all tailored for women attending HIV services in Nairobi, Kenya. Compared to the standard of care, this intervention was associated with a significantly higher likelihood of IPV disclosure, as well as improved mental health, desire to adhere to ART treatment to prevent vertical transmission and eagerness to take actions to improve their home situations [57]. Implementing similar programmes to better identify and support women living with HIV who are experiencing IPV during pregnancy and postpartum could provide benefits for CHEU, but have yet to be tested.

Abuse and separation can often lead to disproportionate burdens on women, leading to financial hardship, childcare responsibility and social stigma [58, 59]. However, separation in abusive relationships may benefit parents and children. Studies from Western nations show that children with separated parents who co‐parent have better neurodevelopmental outcomes than children whose parents stay together with persistent conflict, but this has yet to be studied in the SSA context [60, 61]. Couples affected by HIV separate frequently during pregnancy/postpartum when HIV testing is common. HIV‐serodifferent couples in which the female is living with HIV separate significantly more often than couples in which the male is living with HIV [62]. Paternal involvement can improve birth outcomes, parental satisfaction, maternal engagement with care, and child growth and development [63, 64, 65, 66, 67, 68]. Rwanda's *Sugira Muryango (*“*Strengthen the Family”*), a nationally scaled home‐delivered IPV‐reduction intervention, has successfully reduced acts of abuse, and improved paternal engagement, paternal and maternal mental health, relationship satisfaction and child neurodevelopment [69, 70, 71, 72]. This or similar interventions could be adopted within maternal child health or prevention of vertical transmission programmes in Kenya. Food insecurity has been significantly associated with a greater risk of IPV among Kenyan women living with HIV, as well as a greater risk of preterm birth, which we found to be strongly associated with neurodevelopmental delay among CHEU [73]. Multifactorial interventions to address food insecurity and IPV among pregnant women living with HIV are urgently needed. Ensuring the safety and nutritional health of women and infants during pregnancy and breastfeeding will be paramount to protect CHEU from neurodevelopmental delay and growth faltering [74, 75, 76, 77, 78].

Among all children, regardless of *in utero* HIV exposure, the male sex was associated with lower MDAT scores, a finding not previously observed [41, 42]. Historically, child sex bias has skewed clinical diagnoses for neurodevelopmental disorders and intellectual delays towards boys, most notably in autism spectrum disorder [79]. Research into the mechanistic pathways of such neurodevelopmental sex differences has found complex interactions between different behavioural expression, biologic and environmental factors [80]. Further assessment into possible interactions between male child sex and paternal absence is necessary.

Our study has several strengths, including its large sample size and longitudinal design. This large cohort assessed ART regimen heterogeneity during pregnancy, including the contemporaneous DTG‐based regimens, and is currently undergoing 6‐monthly neurodevelopment assessments until children reach 3 years which will allow for future longitudinal analyses. We conducted the MDAT assessment, which was developed specifically for use in SSA, and relies on both direct child observation and caregiver report. Limitations of the study include the enrolment of infants at 6 weeks of age, which may result in selection bias, as we excluded infants potentially at greatest risk of neurodevelopmental delay (e.g. preterm and/or hospitalised infants, or infants who did not return for their standard 6‐week visit). Additionally, 11% of our study population were lost‐to‐follow‐up by their 12‐month visit; as a result, mother‐infant pairs who were disengaged in routine postnatal care and who could be at elevated risk of poorer outcomes were excluded. Loss‐to‐follow‐up among CHEU in Kenya remains high and is associated with poor child growth and being orphaned, both risk factors for poor neurodevelopment [81]. A small proportion (5%) of our 1‐year‐old CHEU population had pending HIV test results at the time of analysis, posing a risk for misclassification of child HIV status. We anticipate that <1% of these children would be misclassified based on available data for the other 1‐year‐olds in the cohort. Another limitation of our study is that we did not confirm the HIV‐negative status of mothers of CHUU at 1 year postpartum; however, we anticipate few women (∼1%) would have seroconverted between 6 weeks and 12 months postpartum. Our analysis assessed factors collected at baseline to assess associations with 1‐year neurodevelopment; longitudinal analyses using repeated measures on caregiver exposures, such as relationship factors or mental health, are needed to improve the estimation of these associations. The cofactors discussed in this paper were identified through exploratory analyses and may have some degree of false discovery; confirmatory analyses are needed. Some mothers were missing ART data, and analyses assessing ART exposures were only conducted among those with ART data. Our study does not capture mothers <18 years old, mothers not engaged in postnatal care or prevention of vertical transmission programmes and children whose mothers died prior to the child reaching 6 weeks of age. Differential misclassification and recall bias could have influenced findings if mothers with psychological distress and/or mothers living with HIV were more likely to recall factors that influenced their wellbeing and child neurodevelopment. Nurses conducting MDAT assessments were unblinded to maternal HIV status, which could have introduced bias, irrespective of the lack of difference between CHEU and CHUU. The COVID‐19 pandemic led to viral load testing shortages in Kenya, limiting our ability to assess the role of maternal viral load on CHEU neurodevelopment.

## 5 CONCLUSIONS

In this cohort of Kenyan CHEU and CHUU, biologic and social factors were associated with 1‐year neurodevelopment. CHEU and CHUU had similar neurodevelopmental scores across all domains and may be due to the high frequency of DTG use during pregnancy but warrants further investigation. Among CHEU, *in utero* exposure to DTG‐based regimens was associated with higher gross motor scores, compared to EFV‐based regimens. Maternal marital status, father absence, IPV and food insecurity were associated with poorer neurodevelopmental scores. Rigorous longitudinal and mixed methods research are needed to identify modifiable factors among families impacted by HIV and caregiver relationship conflict. It is critical to develop strategies to incorporate neurodevelopmental screening programmes into health systems in SSA with clear referral pathways that equip healthcare workers and caregivers to identify early signs of delays, and to design multi‐factorial interventions that best support children at the highest risk of suboptimal outcomes.

## COMPETING INTERESTS

The authors have no competing interests to disclose.

## AUTHORS’ CONTRIBUTIONS

The initial research question was developed by MAB, GJ‐S, IN and SB‐N. SB‐N, MK, DC, HM, LG, DW, AO, KM, MC and GN conducted and oversaw data collection. MAB and KAL led data management and cleaning. MAB led data analysis and interpretation, with close input from GJ‐S, IN, SB‐N, ADW, JN and DW. The manuscript was first developed by MAB and GJ‐S, and all authors reviewed, contributed to and approved the manuscript for publication.

## FUNDING

The HOPE Study is funded by the National Institutes of Health's National Institute of Child Health and Human Development (4R33HD103079, PI: John‐Stewart), the National Institutes of Health Fogarty International Center (K43TW011422‐01A1, PI: Njuguna) and the National Institute of Mental Health (K01MH121124, PI: Wagner).

## DISCLAIMER

The sponsors did not play any role in the study design, execution of data collection or analysis, manuscript preparation or decision to submit for publication.

## Supporting information


**Table S1**: Maternal ART changes during pregnancy, and median duration of ART use.
**Table S2**: Associations between maternal ART regimen and child neurodevelopment scores, among mothers who started ART pre‐pregnancy.
**Figure S1**: Consort diagram of study participants.

## Data Availability

Data may be made available by authors GJ‐S and SB‐N, upon reasonable request.
