# Supplementary material for: Neurodevelopment of children who are HIV‐exposed and uninfected in Kenya
Source: J Int AIDS Soc. 2023 Nov 1;26(Suppl 4):e26149. doi: 10.1002/jia2.26149 (PMC10618871; doi:10.1002/jia2.26149)
Supplement: Supplementary file 1 — Table S1: Maternal ART changes during pregnancy, and median duration of ART use. Table S2: Associations between maternal ART regimen and child neurodevelopment scores, among mothers who started ART pre‐pregnancy. Figure S1: Consort diagram of study participants. [file JIA2-26-e26149-s001.docx]

**SUPPLEMENTAL APPENDIX**

**Table 1. Maternal ART changes during pregnancy, and median duration of ART use**

| **Maternal ART changes during pregnancy,**  **Among mothers with complete ART data:** | **DTG only**  N = 316 | **DTG from EFV**  N = 129 | **EFV only**  N = 110 | **EFV from DTG**  N = 12 | **Other**  N = 38 |
| --- | --- | --- | --- | --- | --- |
| Initiated ART pre-pregnancy | 240 (76%) | 129 (100%) | 107 (97%) | 12 (100%) | 37 (97%) |
| Duration on ART (months) | 27.4  (14.2, 65.8) | 72.7  (42.0, 98.6) | 65.1  (30.9, 86.2) | 66.9  (52.2, 84.4) | 99.2  (72.1, 135.1) |
| n (%); Median (IQR) | | | | | |

**Table 2. Associations between maternal ART regimen and child neurodevelopment scores, among mothers who started ART pre-pregnancy**

|  | **Social**,  Adjusted coeff (95% CI) | **p** | **Language**,  Adjusted coeff (95% CI) | **p** | **Fine Motor**,  Adjusted coeff (95% CI) | **p** | **Gross Motor**,  Adjusted coeff (95% CI) | **p** |
| --- | --- | --- | --- | --- | --- | --- | --- | --- |
| Most recently prescribed ART regimen during pregnancy (ref: DTG-based) | Ref |  | Ref |  | Ref |  | Ref |  |
| EFV-based | -0.15 (-0.71, 0.40) | 0.59 | 0.14 (-0.17, 0.45) | 0.39 | 0.22 (-0.19, 0.63) | 0.29 | -0.36 (-0.83, 0.10) | 0.13 |
| PI-based | 0.37 (-0.47, 1.20) | 0.40 | 0.17 (-0.30, 0.63) | 0.49 | 0.37 (-0.25, 0.98) | 0.25 | 0.17 (-0.54, 0.87) | 0.64 |
| ART changes during pregnancy (ref: DTG only) | Ref |  | Ref |  | Ref |  | Ref |  |
| DTG, switched from EFV | 0.09 (-0.45, 0.64) | 0.74 | **0.36 (0.05, 0.66)** | **0.02** | -0.09 (-0.49, 0.31) | 0.66 | -0.17 (-0.63, 0.28) | 0.47 |
| EFV only | 0.00 (-0.61, 0.62) | 0.99 | 0.28 (-0.06, 0.62) | 0.15 | 0.27 (-0.18, 0.73) | 0.24 | -0.39 (-0.91, 0.12) | 0.14 |
| EFV, switched from DTG | -0.89 (-2.31, 0.53) | 0.22 | 0.16 (-0.62, 0.95) | 0.76 | -0.33 (-1.37, 0.72) | 0.54 | -0.67 (-1.86, 0.53) | 0.28 |
| Other regimens | 0.42 (-0.44, 1.28) | 0.34 | 0.29 (-0.19, 0.76) | 0.26 | 0.36 (-0.27, 0.99) | 0.27 | 0.11 (-0.62, 0.82) | 0.78 |

**Figure 1. Consort diagram of study participants**

**HOPE Study**

2,000 mother-infant pairs

CHUU enrolled at 6-weeks

(n=1000)

CHEU enrolled at 6-weeks

(n=1000)

CHEU terminated (n=22):

Withdrew (n=3)

Death of child (n=8)

Relocated (n=2)

Child diagnosed with HIV (n=8)

Other (n=1)

CHEU lost to follow up by 1-year (n=112)

*Missing 1-yr data (n=23)*

CHUU terminated (n=26):

Withdrew (n=10)

Death of child (n=14)

Relocated (n=1)

Other (n=1)

CHUU lost to follow up by 1-year (n=114)

*Missing 1-yr data (n=18)*

CHUU who attended 1-year follow up visit

(n=842)

CHEU who attended 1-year follow up visit

(n=843)

CHEU 1-year MDAT assessments

No assessment done (n=107)

Incomplete or invalid assessment (n=24)

Child diagnosed with HIV after 1-uear visit (n=3)

CHUU 1-year MDAT assessments

No assessment done (n=98)

Incomplete or invalid assessment (n=29)

**CHEU included in final analysis**

(n=709)

**CHUU included in final analysis**

(n=715)
